# The Mediating Role of Leisure-Time Physical Activity and Mobility in the Life Satisfaction of Older Adults

**DOI:** 10.1093/geroni/igaf122.2754

**Published:** 2025-12-31

**Authors:** Yongseop Kim, Hyejin Park, Jaehyun Kim, Min Jun Hwang, Junhyoung Kim

**Affiliations:** University of Utah, University of Utah, Utah, United States; University of Utah, Salt Lake City, Utah, United States; East Carolina University, Greenville, North Carolina, United States; Phillips Exeter Academy, Exeter, New Hampshire, United States; Texas A&M University, College Station, Texas, United States

## Abstract

**Background:**

Leisure Time Physical Activity (LTPA) has been associated with numerous health benefits for older adults, including enhanced physical function and life satisfaction. However, older adults often experience physical limitations that hinder participation in LTPA. While prior research has examined the direct effects of LTPA on well-being, the mechanisms underlying this relationship, particularly the mediating roles of mobility and activities of daily living (ADL) performance, remain underexplored.

**Methods:**

This study utilized data from the 2022 National Health Interview Survey (NHIS) (N = 8,779) to examine the relationship between moderate LTPA, mobility, ADL performance, and life satisfaction in older adults. Path analysis was conducted to explore the mediating effects of mobility and ADL performance on the association between LTPA and life satisfaction.

**Results:**

Findings revealed that LTPA participation was significantly associated with improved mobility and ADL performance, both of which were positively linked to life satisfaction. Path analysis demonstrated that mobility and ADL performance mediated the relationship between LTPA and life satisfaction (Estimate = −.010, 95% CI [−.012, −.008]; Estimate = −.004, 95% CI [−.005, −.003]).

**Conclusion:**

This study highlights the importance of promoting moderate LTPA among older adults to enhance physical function and life satisfaction. Given the identified mediating effects of mobility and ADL performance, future interventions should focus on structured LTPA programs that support independence and overall well-being in aging populations. These findings provide valuable insights for policymakers and healthcare professionals in designing effective physical activity interventions for older adults.

